# In silico identification and experimental validation of cellular uptake by a new cell penetrating peptide P1 derived from MARCKS

**DOI:** 10.1080/10717544.2021.1960922

**Published:** 2021-08-02

**Authors:** Linlin Chen, Xiangli Guo, Lidan Wang, Jingping Geng, Jiao Wu, Bin Hu, Tao Wang, Jason Li, Changbai Liu, Hu Wang

**Affiliations:** a Department of Pathology and Immunology, Medical School, China Three Gorges University, Yichang, China; b Hubei Key Laboratory of Tumor Microenvironment and Immunotherapy, China Three Gorges University, Yichang, China; c Affiliated Ren He Hospital of China Three Gorges University, Yichang, China; d The First Clinical Medical College of China Three Gorges University, Yichang, China; e Department of Biology, Johns Hopkins University, Baltimore, MD, USA

**Keywords:** Cell-permeable peptides (CPPs), bioinformatics, plasmid DNA delivery

## Abstract

Viral vectors for vaccine delivery are challenged by recently reported safety issues like immunogenicity and risk for cancer development, and thus there is a growing need for the development of non-viral vectors. Cell penetrating peptides (CPPs) are non-viral vectors that can enter plasma membranes efficiently and deliver a broad range of cargoes. Our bioinformatic prediction and wet-lab validation data suggested that peptide P1 derived from MARCKS protein phosphorylation site domain is a new potential CPP candidate. We found that peptide P1 can efficiently internalize into various cell lines in a concentration-dependent manner. Receptor-mediated endocytosis pathway is the major mechanism of P1 penetration, although P1 also directly penetrates the plasma membrane. We also found that peptide P1 has low cytotoxicity in cultured cell lines as well as mouse red blood cells. Furthermore, peptide P1 not only can enter into cultured cells itself, but it also can interact with plasmid DNA and mediate the functional delivery of plasmid DNA into cultured cells, even in hard-to-transfect cells. Combined, these findings indicate that P1 may be a promising vector for efficient intracellular delivery of bioactive cargos.

## 1. Introduction

Preventive and therapeutic vaccines have promising applications to prevent infection and treat cancer and other health issues globally (Harper et al., 2020). Although several vaccines against human papillomavirus (HPV), viral hepatitis, influenza, measles, varicella, rubella, mumps and rotavirus (Knuf et al., 2008) have been widely used to save millions of lives, vaccines for newly discovered pathogens like the respiratory virus COVID 19 are urgently needed (Du et al., 2018). Since COVID-19’s emergence and spread, academic institutions and pharmaceutical companies have raced to discover and develop COVID-19 vaccines, including protein, DNA, and mRNA-based vaccines. Although the antigen and adjuvant design, as well as immunization method, can directly affect a vaccine’s potency and duration of its efficacy, effective intracellular delivery approach and platform are also vital for the vaccine to achieve robust humoral and cellular immunity.

In general, delivery systems used to transfer biological molecules into cells, including physical tools (Liu et al., 2016; Du et al., 2018), liposomes (Yu et al., 2019), polymers (Bose et al., 2019), and nanoparticles (Garg and Dewangan, 2020), are widely used in laboratory investigations, but these delivery systems are not as clinically effective (Ain et al., 2020). Although viral vectors are efficient for delivering vaccine content into host cells, they are challenged by recently reported safety issues like immunogenicity and risk for developing cancer (Batty and Lillicrap, 2019), thus resulting in the need for and development of non-viral vectors for vaccine delivery.

Cell penetrating peptides (CPPs, 5–50 amino acids) are short peptides that can enter the plasma membrane efficiently and deliver a broad range of cargoes, including nucleic acids, proteins, peptides, and nanoparticles, into cells (Liu et al., 2016; Wu et al., 2018). A large number of CPPs have been identified, but some of them have shown low cellular uptake (Wang et al., 2010; Gautam et al., 2015; Liu et al., 2016). Therefore, identification of novel CPPs for therapeutics’ delivery is still an urgent affair.

Numerous studies have suggested that applying bioinformatic tools for CPP predictions prior to wet-lab experimental characterization can save time and money (Arif et al., 2020; Kardani and Bolhassani, 2021). Therefore, in this study, we combined bioinformatic prediction and experimental validation to find and characterize novel and potent CPPs as a DNA vaccine and drug delivery system. Here, we identified a novel CPP named P1 that is derived from the fragment of MARCKS. Predictions of P1’s physical-chemical properties, structures, and penetration properties were performed through *in silico* approaches. Through wet-lab experimental validation, P1’s penetration efficiency, mechanism of penetration, and in-vitro cytotoxicity assay results were also studied to further confirm its penetration ability. Lastly, we found that P1 can introduce plasmid DNA into cultured cell lines, even in hard-to-transfect cells. These findings combined indicate that P1 may be a promising vector for efficient intracellular delivery of bioactive cargos.

## 2. Materials and methods

### 2.1. Peptide, cell line, and cell culture

Fluorescein isothiocyanate (FITC) conjugate to the N-terminus of synthetic P1 (FITC-(Acp)-KKKKKRFSFKKSFKLSGFSFKKNKK) was customly synthesized using f-moc solid-phase synthesis and further assayed using reversed-phase analytical high-performance liquid chromatography at >96% purity by China Peptides (Shanghai, China). NCO control peptide (Wang et al., 2016; Geng et al., 2020), TAT (Wang et al., 2010; 2016; Ding et al., 2019) and MT23 (Zhou et al., 2017) were fluorescently labeled with FITC by the same company as P1 shown above. Lyophilized peptides and conjugates were dissolved in phosphate buffered saline (PBS) and stored at −20 °C unless otherwise stated.

Human breast cancer cell line MCF7, human non-small cell lung cancer cell line A549, mouse microglial BV2 cell line, and rat hepatic stellate cell line T6 were maintained in Dulbecco’s modified Eagle’s medium (DMEM) supplemented with 10% heat-inactivated fetal bovine serum (FBS) and 1% penicillin (100 U/ml)–streptomycin (0.1 mg/ml), and the cells were grown in humidified incubators at 37 °C and 5% CO_2_.

### 2.2. Physical-chemical predictions of P1

The following physico-chemical properties of P1 including Accessible Residues, Hphob./Kyte & Doolittle, Hphob./Eisenberg et al., Average Flexibility, Bulkiness, Polarity/Grantham, and Relative mutability were calculated by protscale tool from ExPASy (https://web.expasy.org/protscale/) (Wilkins et al., 1999). The intrinsic disorder parameters of peptide P1 were also predicted by IUPred2A (http://iupred2a.elte.hu) (Mészáros et al., 2018), PrDOS (http://prdos.hgc.jp/cgi-bin/top.cgi) (Ishida & Kinoshita, 2007), and ANCHOR2 (http://anchor.elte.hu/) (Mészáros et al., 2018).

### 2.3. Modeling the 3 D structure of peptide P1

Amino acid sequence of P1 was submitted to RaptorX web server (http://raptorx.uchicago.edu/) (Wang et al., 2016) and I-TASSER (Iterative Threading ASSEmbly Refinement, https://zhanglab.ccmb.med.umich.edu/I-TASSER/) online server (Yang and Zhang, 2015). The three-dimensional (3 D) structure of P1 was generated by I-TASSER. PROCHECK (Laskowski et al., 1993), ERRAT (Colovos & Yeates, 1993) and Verify-3D (https://saves.mbi.ucla.edu/) (Eisenberg et al., 1997) were used to validate the quality of predicted models. Moreover, Ramachandran plot (Ramachandran et al., 1963) was used to analyze conformational regions of predicted structure. Lipid membrane interaction with peptide was predicted by PPM server (https://opm.phar.umich.edu/ppm_server) (Lomize et al., 2012), CELLPM Server (https://cellpm.org/cellpm_server) (Lomize & Pogozheva, 2018) and TMHMM server (http://www.cbs.dtu.dk/services/TMHMM/) (Möller et al., 2001).

### 2.4. Determination of toxicity, allergenicity, hemolytic potency, and half-life

IEDB Immunogenicity Predictor (http://tools.iedb.org/immunogenicity/) was used to assess the immunogenicity of CPPs (Calis et al., 2013). AllerTop web server (https://www.ddg-pharmfac.net/AllerTOP/) (Dimitrov et al., 2014) and AllergenFP (http://ddg-pharmfac.net/AllergenFP) web server (Dimitrov et al., 2014) were used to investigate allergenicity of candidate CPPs. ToxinPred web server (https://webs.iiitd.edu.in/raghava/toxinpred/algo.php) was used to investigate toxicity of CPPs. ProtLifePred web server (http://protein-n-end-rule.leadhoster.com/) (Bachmair et al., 1986) was used to calculate half-life in *E.coli* and mammalian cells. Furthermore, HemoPI web server (https://webs.iiitd.edu.in/raghava/hemopi/design.php) (Chaudhary et al., 2016) was used to predict hemolytic property of CPP candidate.

### 2.5. Penetration prediction of peptide P1

Penetration properties of MARCKS gene family from different species were predicted using CPPred-RF (http://server.malab.cn/CPPred-RF/) (Wei et al., 2017), C2Pred (http://lin.uestc.edu.cn/server/C2Pred) (Tang et al., 2016), MLCPP (http://www.thegleelab.org/MLCPP/) (Manavalan et al., 2018), SkipCPP-Pred (http://server.malab.cn/SkipCPP-Pred/Index.html) (Wei et al., 2017) and CellPPD (http://crdd.osdd.net/raghava/cellppd/) (Gautam et al., 2013). Predicted results from these web servers, including probability score or confidence, were analyzed using GraphPad Prism version 7.00.

### 2.6. Cellular uptake and fluorescent microscopy

Cells were suspended in regular culture media and seeded on coverslip in a 24-well plate at a concentration of 1.6 × 10^4^ cells/well. Rinsing with PBS twice after 24 h of incubation for cell attachment, FITC-labeled peptides at indicated concentrations were added with 0.5 ml serum-free media/well and incubated for 1 hour at 37 °C. After incubation, the cells were washed with PBS at least three times. 4′,6-diamidino-2-phenylindole (DAPI) was added to each well and incubated for 5 min after fixation with 4% paraformaldehyde (PFA) in PBS. The cells were washed with PBS, mounted on the slide, and observed by fluorescence microscopy (Nikon, Tokyo, Japan).

To analyze the internalization efficiency of CPP, the fluorescence of cell lysates and supernatants was measured using TECAN Safire multi-well reader with excitation and emission wavelengths set to 485 nm and 535 nm, following the protocol published (Wang et al., 2010; Ma et al., 2015; Wang et al., 2016; 2016; Zhou et al., 2017; Shao et al., 2016; Zhang et al., 2019; Geng et al., 2020). After the cell incubation with peptide and washing step described above, cells were lysed with 300 µl lysis buffer (0.1 M NaOH) for 10 minutes and centrifuged at 800 rpm for 5 minutes. Fluorescence in the 50 µl supernatant transferred to a 96-well plate was monitored by the plate-reader spectrophotometer (Tecan, Mannedorf, Switzerland). The amount of FITC-labeled peptides internalized were normalized by protein concentration, and the experiments indicated were repeated at least three times.

### 2.7. Cytotoxicity Assay

Cytotoxicity was measured using MTT assay. Grown cells were inoculated for 24 h at a density of 10000 cells/well in 96-well plates. After washing with PBS, the indicated concentrations of peptides were added, following by further incubation for 24 h or 48 h. After at least two-times washing, 5 mg/ml MTT dissolved in PBS with serum-free media was added into plates for another 4 h incubation. Dimethyl sulfoxide (DMSO) was added into plates and incubated at 37 °C for 15–30 minutes to dissolve the formazan crystals. The absorbance of aliquot from each well was measured at 490 nm using a Multiskan Spectrum (Thermo Fisher Scientific, Waltham, MA, USA) microplate reader. Experiments were repeated three times.

### 2.8. Hemolytic activities

Mouse erythrocytes free of plasma components were separated by centrifugation at 1000 rpm for 5 minutes and washed three times with PBS. The washed mouse erythrocytes were resuspended in PBS and treated with indicated concentrations of peptides at 37 °C for 2 h. Hemolysis was examined by measuring the absorbance of supernatant at the wavelength of 450 nm, and 0.1% Triton X-100 was used as a positive control.

### 2.9. Gel shift assay

Gel shift assay was performed following the protocol described (Ding et al., 2019; Zhang et al., 2019; Geng et al., 2020). 1 μg of plasmid pdsRed DNA was mixed with serial N/P (nitrogen to phosphate) ratios of peptides, and the mixture was incubated for 30 min at room temperature. After adding 3 μL of loading buffer, DNA migration was measured by using 1% agarose gel electrophoresis. Imaging was performed using Kodak Gel Logic 2200 Imaging System.

To determine the stability of peptide/pDNA complex, 50% serum was added into the mixture for another 4 h. Gel retardation was visualized using a gel imaging system.

### 2.10. Size measurement

For particle size measurement, previous published protocol (Ding et al., 2019) was followed. The P1/pDNA complexes were mixed according to serial N/P ratios. The average particle size of the peptide/pDNA complexes were examined by Zetasizer (Zetasize-Nano ZS90; Malvern Instruments, Worcestershire, UK) and data analysis was performed with Zetasizer software 6.30.

### 2.11. Peptide-mediated transfection

HSC-T6, BV2 and MCF7 cells (4 × 10^4^ cells/well) were seeded onto 24-well plates 24 h before the experiment. Cells were incubated with CPP-P1/pDNA complexes at indicated N/P ratios for 4 hrs in serum-free media, and then were incubated for 24 h or 48 h after adding media with 10% serum. Live cell imaging under fluorescence microscope was carried out to estimate peptide-based transfection efficiency. Cells transfected with TurboFectin (OriGene, Beijing, China) were used as positive transfection reagent.

### 2.12. Statistical analysis

All results are expressed as means ± standard error of the mean (SEM). Significance analysis was performed using GraphPad software Prism 7.0 (GraphPad Software, San Diego, CA, USA). Differences of *p* < 0.05 were considered statistically significant.

## 3. Results

### 3.1. Cpp-P1 identification

Intracellular myristoylated alanine-rich C kinase substrate (MARCKS) protein (87-kDa) is a ubiquitous, highly conserved protein in vertebrates, and its functions include sequestering phosphatidylinositol 4,5-bisphosphate (PIP2) and regulating phospholipase C signaling. To study the conservation of MARCKS phosphorylation site domain (PSD) families, we collected from public sources a total of 116 eukaryotic species (Figure S1(A)). For these PSD families, the protein sequences were aligned (Figure S1(A)), and sequence logo describing amino acid enrichment was illustrated (Figure S1(A)). Phylogenetic tree analysis of those PSD from 116 species was obtained (Figure S1(B)).

Based on the rich lysine residues characterized in MARCKS PSD, we speculate that MARCKS may have CPP penetration property, because a common feature of all CPPs is the richness in positively charged residues. Before conducting more expensive wet-lab testing, we used published web servers to predict MARCKS’s cell-penetrating property. First, we used CellPPD web server to predict whether MARCKS are CPP by using SVM + Motif based method (Figure S2(A)). We also used other published web servers (CPPred-RF, MLCPP, SkipCPP-Pred and C2Pred) to further confirm (Figure S2(B)). Then, we combined the information from these data sets and followed analytical pipeline shown in Figure S2(C) to create a high-efficiency screening method for identifying new CPP. Furthermore, we also compared the prediction confidence of CPP or non-CPP and uptake efficiency between published CPPs, including TAT, hPP3 and MT23. Although the CPP or non-CPP prediction confidence of peptide P1 is not the highest one among these 4 CPPs, predicted uptake efficiency is the highest one (Figure S3). At the end, we found peptide P1 (KKKKKRFSFKKSFKLSGFSFKKNKK) to be the best candidate with low immunogenicity and toxicity.

### 3.2. Physicochemical properties and structure prediction of peptide P1

The physicochemical characterization of peptide P1 was performed using the Protscale software tool in ExPASy to analyze hydrophilicity, bulkiness, accessibility, polarity, flexibility, and mutability. These parameters are represented by the scores shown in Figure S4. Kyte & Doolittle and Eisenberg scale (Figure S4(A)) was used to detect the hydrophilicity of peptide P1, and the results show that the hydrophilicity values (Figure S4(A)) are between −2 (position 5) and 0.5 (position 16). The accessibility and buried values (Figure S4(B)) are between 6 (position 16) and 8 (position 5). The polarity values (Figure S4(C)) are between 8 (position 16) and 10 (position 5), while the bulkiness values (Figure S4(C)) are between 14 (position 20) and 16 (position 11). The average flexibility values (Figure S4(D)) are between 0.415 (position 11) and 0.45 (position 8). The relative mutability values (Figure S4(E)) are between 60 (position 5) and 75 (position 20). Moreover, the disorder probability of each amino acid predicted by IUPred2A, ANCHOR2 and PrDOS (Figure S4(F)) suggests that disorder regions may be located at the two ends of peptide P1.

**Figure 1.**
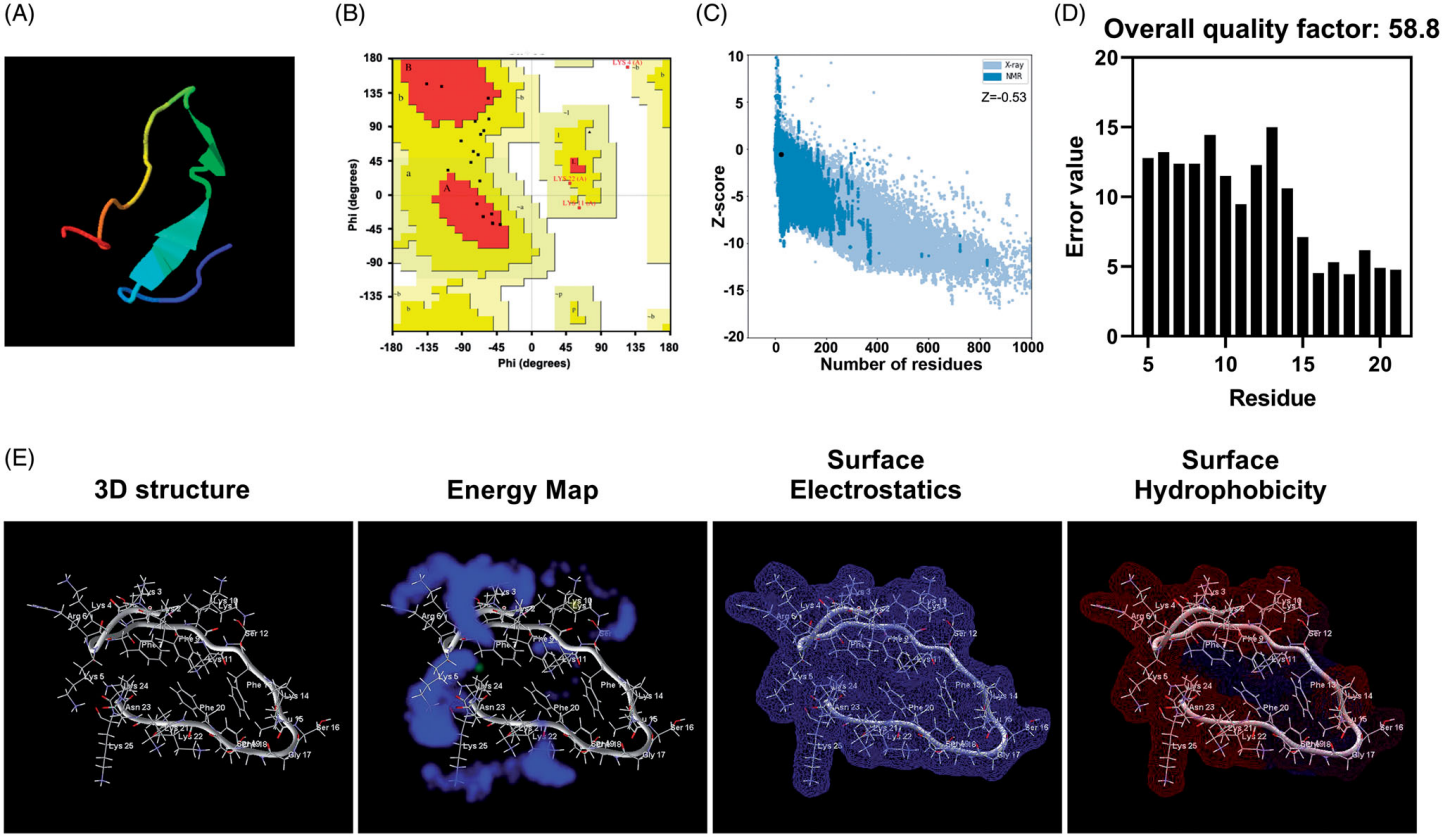
Peptide P1 secondary structure prediction. (A) Three-dimensional structure model of peptide P1 predicted by I-TASSER. (B) I-TASSER predicted peptide P1 structure validation of the assessed Ramachandran plot. (C) I-TASSER predicted peptide P1 structure evaluated by ProSA-web. (D) Overall quality of I-TASSER predicted P1 structure evaluated by ERRAT server. (E) Three-dimensional structure, energy map, surface electrostatics, and hydrophobicity representation of peptide P1.

Residue-residue contact map prediction can be used to represent the probability of a three-dimensional peptide structure for all possible pairs of amino acid residues. Here, the most probable contact was found between Phe-9 and Phe-20 (Figure S5(A)). Then, RaptorX server was used to predict solvent accessibility (Figure S5(B), top panel), 3-class secondary structure (Figure S5(B), middle panel) and 8-secondary structure (Figure S5(B), bottom panel) of peptide P1. Residues 18 to 22 of peptide P1 have a higher probability to form beta-sheet but a relatively lower probability of alpha-helix. Helical wheel projection of peptide P1 was obtained from Heliquest web server (Figure S5(C)), and hydrophobic face was illustrated with hydrophobic residues (Leu and Phe). Tertiary structural construction was also performed using I-TASSER online server. Figure 1(A) presented the best model generated (TM-score = 0.45 ± 0.14, C-score=−2.29 and RMSD = 5.8 ± 3.6 Å), and the quality of this model was validated by Ramachandran plot. 40.9% residues of modeled peptide P1 are in the most favored region, and 54.6% are in additional and generously allowed region (Figure 1(B)). Then, ProSA web server was used to calculate the Z score (quality score), which should fall in a characteristic range (Figure 1(C)). Additionally, ERRAT server predicted plot in Figure 1(D) displayed an overall quality factor reaching 58.8. Three-dimensional structure of peptide P1, including energy map, surface electrostatics, and surface hydrophobicity, are graphically presented in Figure 1(E).

### 3.3. Penetration properties and immunogenicity prediction of peptide P1

**Figure 2.**
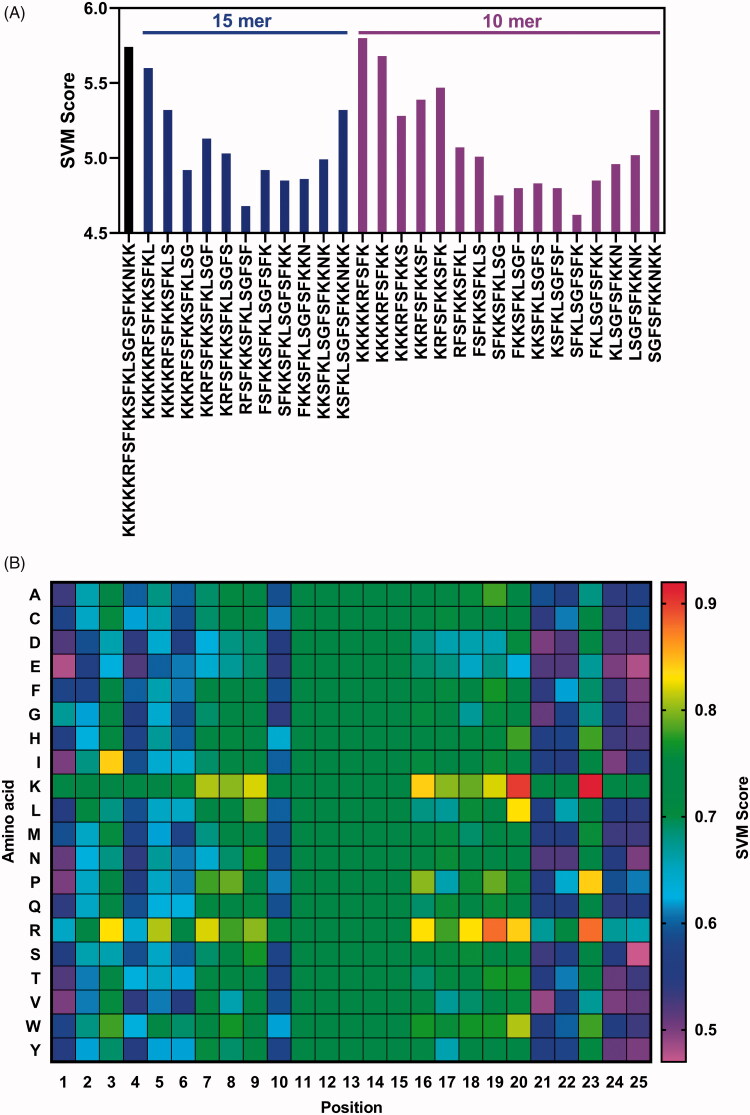
Core motif identification of peptide P1. (A) Penetration property prediction of peptide P1 with 15-mer and 10-mer truncation analysis. (B) The heatmap (SVM score) representing penetration property of peptide P1 with single mutation predicted by CellPPD.

Previous studies have suggested that uptake efficiency of CPPs are correlated with sequence length and basic residue (arginine or lysine) positions (Futaki et al., 2007; Liu et al., 2016; Yadahalli & Verma, 2020). To further evaluate whether peptide P1 is sensitive to changes in amino acid sequences, peptide truncation (Figure 2(A)) and single mutation (Figure 2(B)) prediction by CellPPD were performed. Truncation analysis in Figure 2(A) suggested that 15-mer and 10-mer truncated peptide P1 fragments have significantly decreased penetration property, although the first 10-mer of N-terminal peptide P1 still had a higher score than the full-length peptide P1, which may be due to the core motif that determines the penetration property of peptide P1. Moreover, we also performed penetration property prediction of peptide P1 with single mutation in different sites. The heatmap of Figure 2(B) suggests that the penetration property of peptide P1 is not determined by the positions from 11 to 20. These data suggested that penetration property of peptide P1 is sequence-length dependent, and its N-terminal and C-terminal are crucial for its penetration.

### 3.4. Penetration property validation

**Figure 3.**
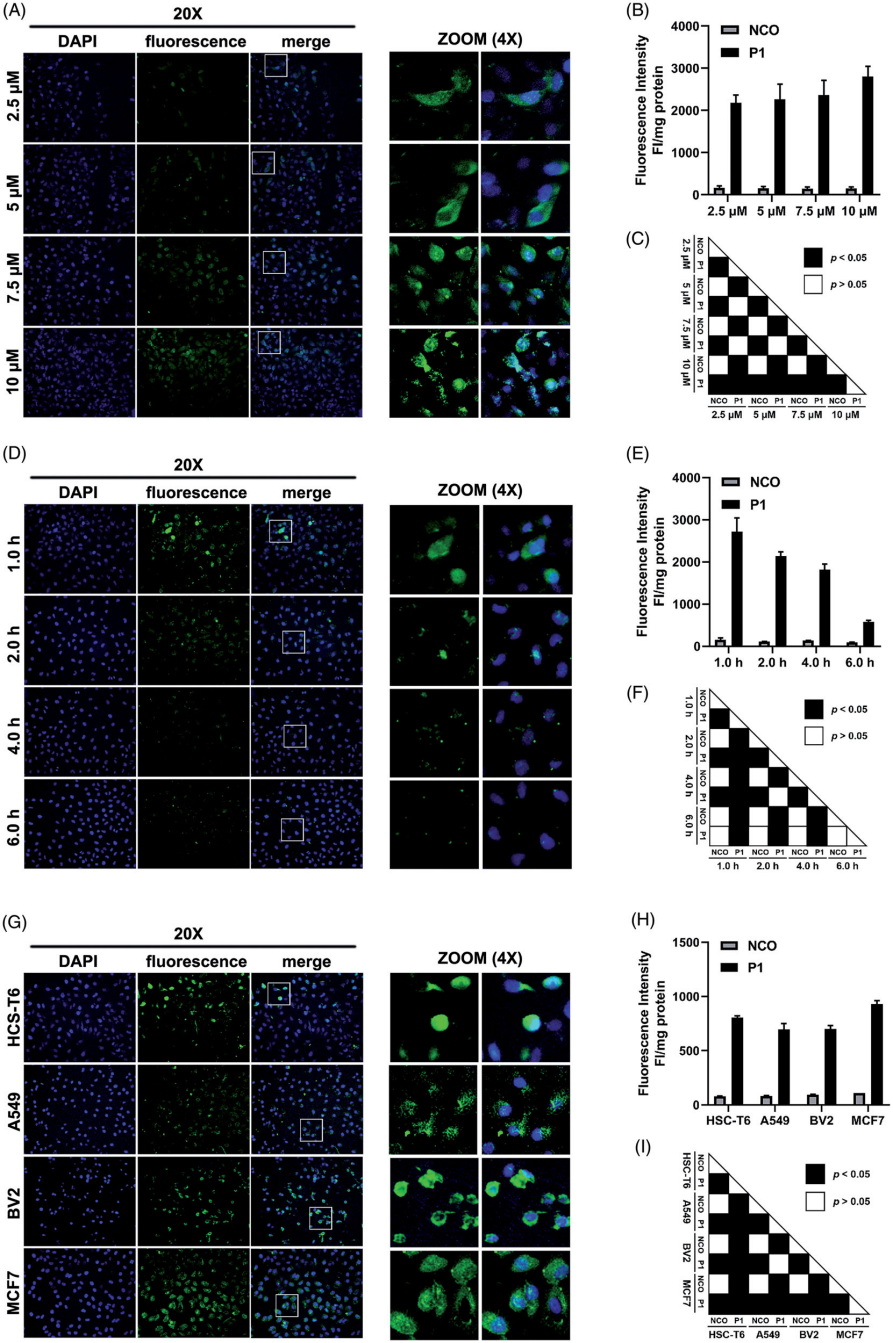
Penetration property validation. (A) Fluorescence microscopy images of peptide P1 penetration at indicated peptide concentration. White box indicated 4 times zoom region shown in the right panel. (B) Quantification of fluorescent intensity of peptide P1 penetration at indicated peptide concentration. The fluorescence of the cellular uptake was normalized by cellular protein. Values represent mean ± SEM. (C) The corresponding *p*-value plot between data pairs presenting in (B). The one-way analysis of variance (ANOVA) was used to compare the differences between the control and experimental values. (D) Fluorescence microscopy images of peptide P1 (5 μM) penetration with different incubation times. White box indicated 4 times zoom region shown in the right panel. (E) Quantification of fluorescent intensity of peptide P1 (5 μM) penetration with different incubation times. The fluorescence of the cellular uptake was normalized by cellular protein. Values represent mean ± SEM. (F) The corresponding *p*-value plot between data pairs presenting in Figure 3(E). ANOVA was used to compare the differences between the control and experimental values. (G) Fluorescence microscopy images of peptide P1 (5 μM) penetration in different cell lines. White box indicated 4 times zoom region shown in the right panel. (H) Quantification of fluorescent intensity of peptide P1 (5 μM) penetration in different cell lines. The fluorescence of the cellular uptake was normalized by cellular protein. Values represent mean ± SEM. (I) The corresponding *p*-value plot between data pairs presenting in Figure 3(H). ANOVA was used to compare the differences between the control and experimental values.

After in-silico prediction, we also conducted wet-lab validation of penetration property of peptide P1. HSC-T6 cells were incubated with indicated concentration of FITC-labeled peptide P1 for 1 hour. Fluorescence microscopy (Figure 3(A)) and fluorescence intensity (Figure 3(B,C)) normalized with protein concentration suggested that the penetration property of peptide P1 is concentration dependent. Then, after 1 hour treatment, we found that the fluorescence of peptide P1 was significantly decreased as incubation time increased in both fluorescent images (Figure 3(D)) and fluorescence quantification (Figure 3(E)). Moreover, we investigated the penetration efficiency of peptide P1 in different cell lines. Although we found that peptide P1 had a higher penetration efficiency in MCF7, peptide P1 could enter into hard-to-translocate BV2 cells in significant amount as shown in fluorescent images (Figure 3(E)) and fluorescence quantification (Figure 3(F)).

### 3.5. Penetration efficiency affected by penetration enhancer and penetration efficiency comparison of different peptides

**Figure 4.**
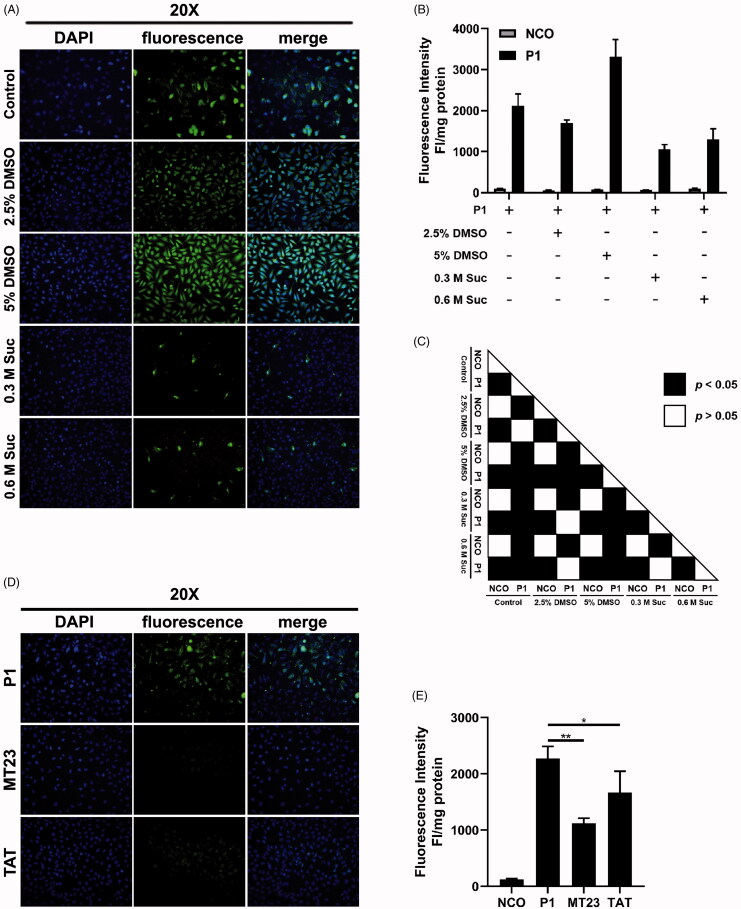
Peptide P1 penetration comparison between penetration enhancer and peptide. (A) Fluorescence microscopy images of peptide P1 (5 μM) incubated with different penetration enhancers. (B) Quantification of fluorescent intensity of peptide P1 (5 μM) incubated with different penetration enhancers. The fluorescence of the cellular uptake was normalized by cellular protein. Values represent mean ± SEM. (C) The corresponding *p*-value plot between data pairs presenting in Figure 4(B). ANOVA was used to compare the differences between the control and experimental values. (D) Fluorescence microscopy images of peptide P1 (5 μM) and other published CPPs. (E) Quantification of fluorescent intensity of peptide P1 (5 μM) and other published CPPs. The error bars express SEM, the fluorescence of the cellular uptake was normalized by cellular protein. ANOVA was used to compare the differences between the control and experimental values, * indicated *p* < .05, and ** indicated *p* <.01.

Previous studies have suggested that DMSO and sucrose can enhance the penetration efficiency of well-known CPP, such as TAT (Wang et al., 2010; 2016), hPP3 (Shao et al., 2016), hPP10 (Wang et al., 2016), MT23 (Zhou et al., 2017), Scp01-b (Zhang et al., 2019) and Dot1l (Geng et al., 2020). To further confirm the enhancement of penetration enhancer on peptide P1, we examined the penetration efficiency of peptide P1 incubated with 2.5% and 5% DMSO, and 0.3 M and 0.6 M sucrose. We found that the penetration efficiency of peptide P1 was only enhanced by 5% DMSO treatment and was inhibited by sucrose both in fluorescent microscopy (Figure 4(A)) and quantification (Figure 4(B)). Besides, reports have shown that some chemical agents like chloroquine (CQ) that enable the escape of CPP from endosomal entrapment (Shiraishi and Nielsen, 2006). Therefore, we tested the effect of penetration efficiency of P1 incubated with 100 μM CQ. Our data indicated that CQ treatment had no obvious effect on the penetration efficiency of P1 in fluorescent quantification (Figure S6). Additionally, we also compared the penetration property of peptide P1 with MT23 and TAT, and we found that peptide P1 has a higher penetration efficiency than MT23 and TAT (Figure 4(D,E)).

### 3.6. Penetration mechanism of peptide P1

**Figure 5.**
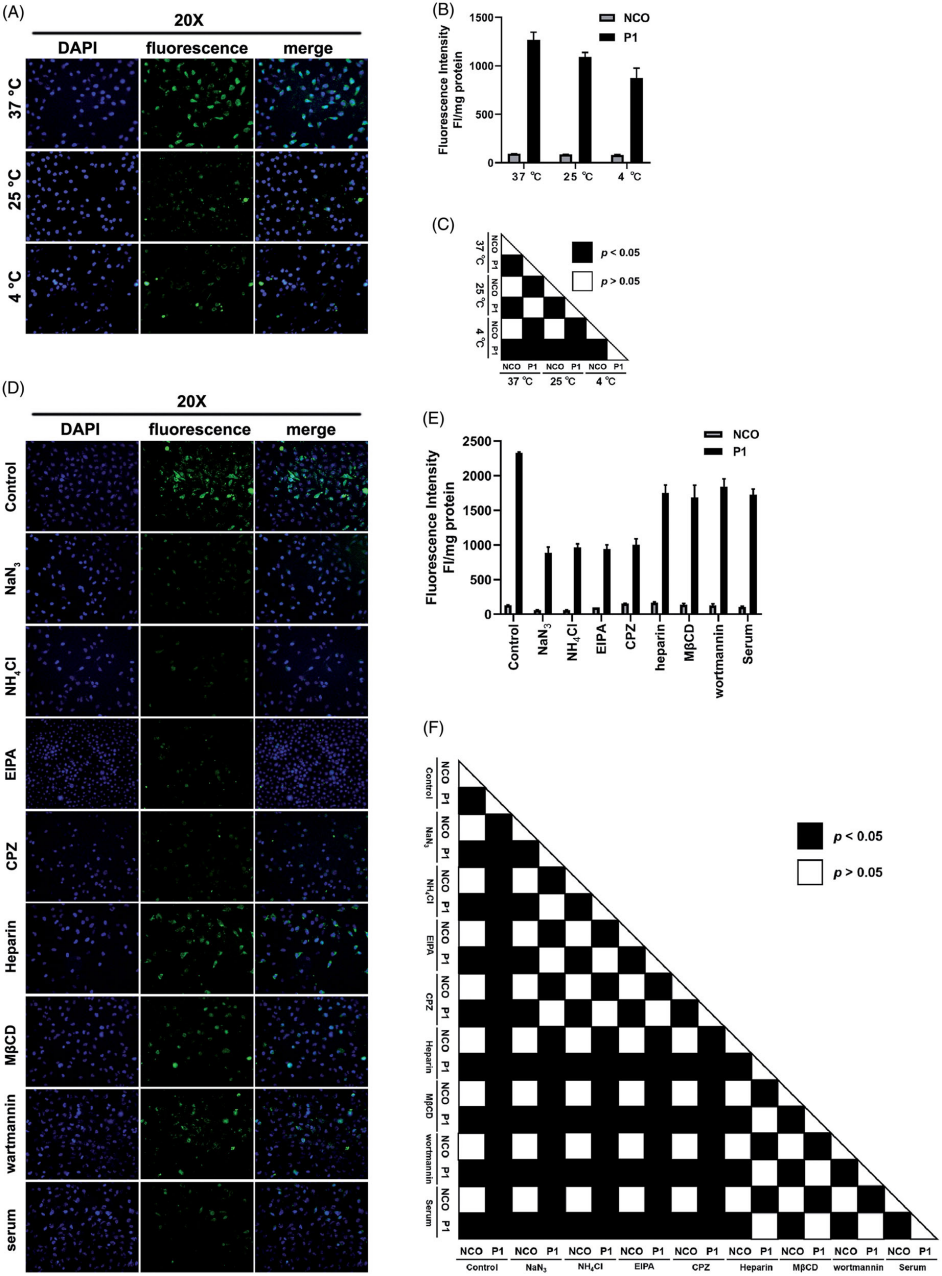
Mechanisms involved in peptide P1 penetration. (A) Fluorescence microscopy images of peptide P1 (5 μM) incubated at different temperatures. (B) Quantification of fluorescent intensity of peptide P1 (5 μM) incubated at different temperatures. The fluorescence of the cellular uptake was normalized by cellular protein. Values represent mean ± SEM. (C) The corresponding *p*-value plot between data pairs presenting in (B). ANOVA was used to compare the differences between the control and experimental values. (D) Fluorescence microscopy images of peptide P1 (5 μM) incubated with different endocytosis inhibitors. (E) Quantification of fluorescent intensity of peptide P1 (5 μM) incubated with different endocytosis inhibitors. The fluorescence of the cellular uptake was normalized by cellular protein. Values represent mean ± SEM. (F) The corresponding *p*-value plot between data pairs presenting in (E). ANOVA was used to compare the differences between the control and experimental values.

To directly investigate the mechanism behind intracellular uptake of peptide P1, we first examined the penetration ability of peptide P1 in HSC-T6 cells at different temperatures. Penetration efficiency of peptide P1 at 25 °C and 4 °C was significantly inhibited compared to normal temperature at 37 °C in both fluorescence microscopy images (Figure 5(A)) and fluorescence intensity quantifications (Figure 5(B)). Moreover, when endocytosis inhibitors sodium azide (mitochondrial electron transport chain uncoupler (Wang et al., 2010)), ammonium chloride (endo-lysosomal acidification neutralizer (Qiang et al., 2018)), 5-(N-ethyl-N-isopropyl) amiloride (EIPA, inhibitor of macropinocytosis (Elmquist et al., 2006)), chlorpromazine (CPZ, inhibitor of clathrin-mediated endocytosis (Park et al., 2019)), heparin (soluble analogue of heparan (Wang et al., 2016; Geng et al., 2020)), MβCD (inhibitor of lipid raft-mediated endocytosis (Zhang et al., 2019)), and Wartmannin (inhibitor of receptor mediated endocytosis by blocking PI-3 kinase (Geng et al., 2020)) were used, penetration efficiency of peptide P1 was significantly reduced (about 55% decreased), and the uptake of peptide P1 was inhibited (by about 20%) (Figure 5(C,D)). This data suggested that penetration of peptide P1 involves the endocytosis pathway. Although these data are consistent with low temperature conditions, P1 can still efficiently penetrate the membrane and enter the cell. Thus, direct penetration is still a major possible non-endocytic pathway mediating cellular uptake of peptide P1, endocytic pathway partially mediate translocation P1 as well.

### 3.7. Cytotoxicity and safety evaluation of peptide P1

**Figure 6.**
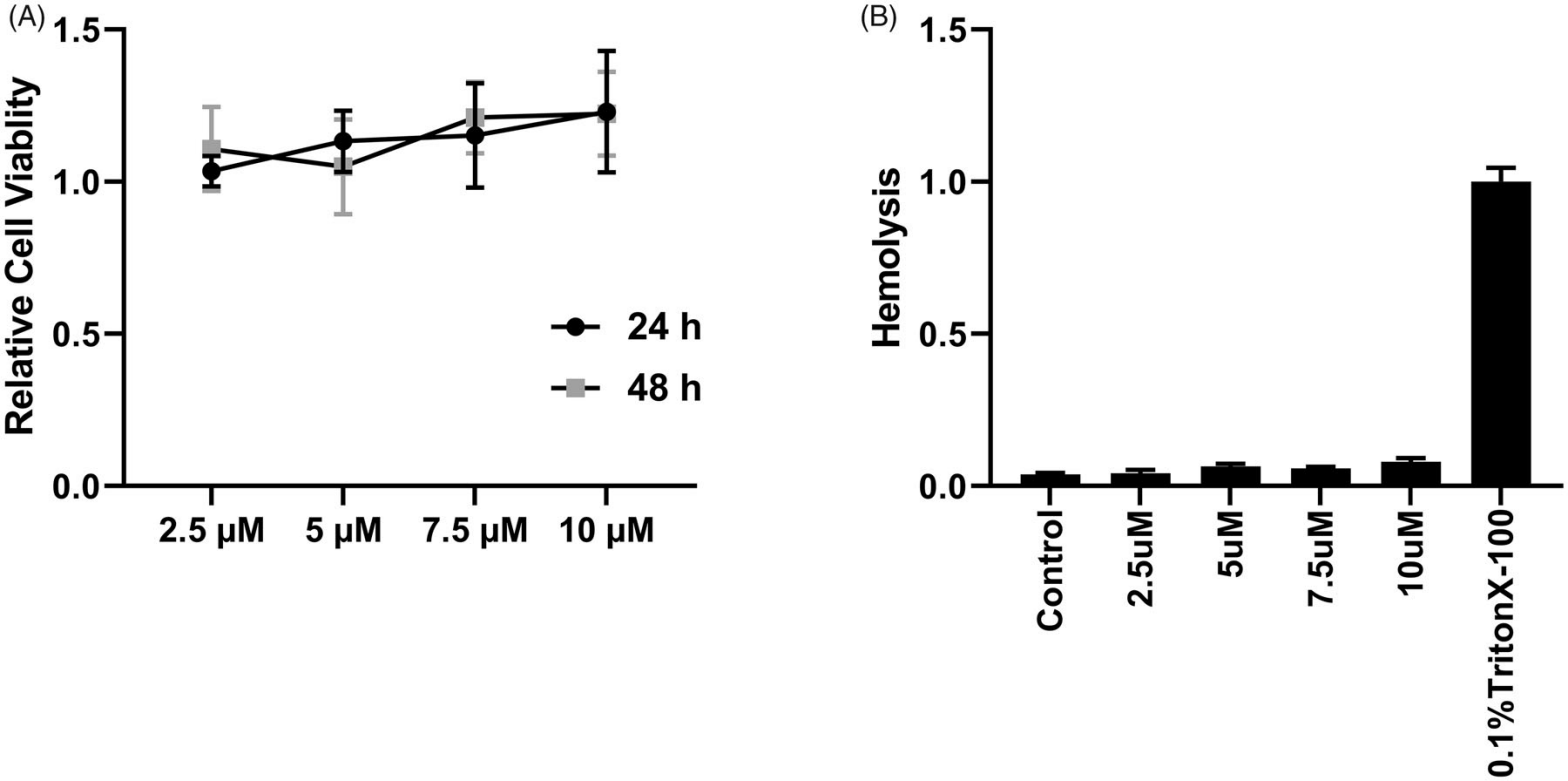
Cytotoxicity and hemolysis evaluation. (A) MTT assay of peptide P1 incubation in HSC-T6 cells with indicated concentration at 24 or 48h. (B) Murine red blood cell hemolysis of peptide P1 with indicated concentration. Values represent mean ± SEM. ANOVA was used to compare the differences between the control and experimental values, **** indicated *p* < .0001.

Before we performed the cell-based cytotoxicity assay, we conducted bioinformatic assay to predict the immunogenicity of peptide P1, and we found that Class I Immunogenicity of peptide P1 is much low than other peptides, such as hPP3, hPP10, MT23, Dot1l (which we previously identified), and classical CPP-TAT (Figure S7(A)). Furthermore, in MTT assay, we did not find significant cytotoxicity at indicated concentration of peptide P1 after 24 h and 48 h treatment in HSC-T6 cells (Figure 6(A)). Then, HemoPI was used to predict whether peptide P1 can affect the integrity of red blood cells. Red blood cells were minimally perturbed by peptide P1 from bioinformatic prediction (Figure S7(B)) and hemolysis assay from wet-lab validation (Figure 6(B)). In addition, toxicity, allergenicity, and half-life prediction were conducted, and we found that peptide P1 is a non-toxin, non-allergen peptide and has the same half-life as our previously identified peptides (Figure S7(C)). Lastly, we also performed membrane interaction (Figure S8) prediction on peptide P1 and Dot1l. Residue 15 of peptide P1 may embed into lipid bilayers, as predicted by PPM server modeling (Figure S8(A)) and CellPM server (Figure S8(B)), and the peptide P1 has low water-to-DOPC bilayer transfer energy ΔG(z) compared with Dot1l (Figure S8(C)). Lastly, we also conducted TMHMM prediction for cellular localization by calculating the probability of binding a peptide to the cell membrane (Figure S9). TMHMM prediction data of peptide P1 (Figure S9(A)), Dot1l (Figure S9(B)), hPP3 (Figure S9(C)), hPP10 (Figure S9(D)), TAT (Figure S9(E)) and MT23 (Figure S9(F)), as well as quantification of total probability of these peptides shown in Figure S9(G), suggest that peptide P1 have nearly the same membrane interaction probability as the other peptides.

### 3.8. Peptide P1 mediated plasmid delivery

**Figure 7.**
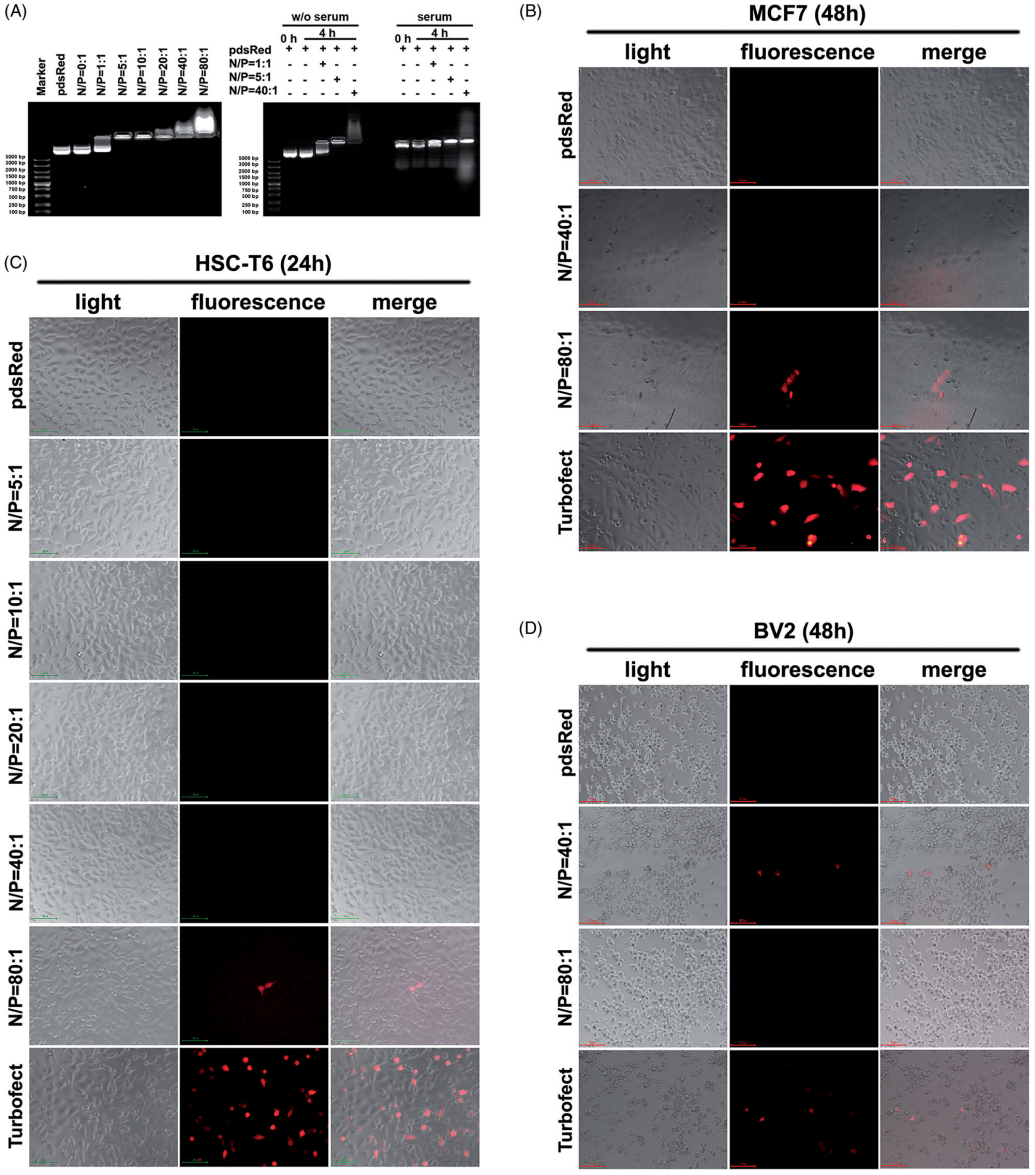
Peptide P1 medicates plasmid delivery in vitro. (A) Agarose gel shift assay on different N/P ratios (left panel), and peptide/pDNA stability in serum for 4 h. (B) Fluorescence microscopy of RFP expression in MCF7 cell cultured for 48 h. (C) Fluorescence microscopy of RFP expression in HSC-T6 cell cultured for 24 h. (D) Fluorescence microscopy of RFP expression in BV2 cell cultured for 48 h.

Our data shown above suggest that peptide P1 is cell penetrating peptide which can enter into the cells efficiently, but its ability to mediate cargo delivery into cells is still unknow. Therefore, we examined the cargo delivery property of peptide P1 in mediating plasmid DNA transfection. Results shown in Figure 7(A) (left panel) suggests that peptide P1 can interact with plasmid DNA pdsRed and form complex at the N/P ratio of 5:1, and the peptide/pDNA complex was still stable in serum (Figure 7(A), right panel). The particle size of the P1 peptide/pDNA complex was larger than that of plasmid DNA alone, and the diameter of the complex was increased as N/P ratio increase (between 600 nm and 1100 nm) (Figure S10). Furthermore, we added these peptide/pDNA complexes in MCF7 (Figure 7(B)), HSC-T6 (Figure 7(C)) and hard-to-transfect cell line BV2 (Figure 7(D)) for 24 h or 48 h incubation and examined red fluorescent protein (RFP) expressions under fluorescence microscope. We found that peptide P1 can mediate pdsRed plasmid transfection at the N/P ratio of 80:1 in MCF7 (Figure 7(B)) and HSC-T6 cells (Figure 7(C)). Interestingly, we observed RFP expression in BV2 cells to be at the N/P ration of 40:1, although we could not find RFP positive cells in the 80:1 group (Figure 7(D)). These data suggest that peptide P1 not only can penetrate into cells by itself but also can mediate plasmid delivery *in vitro*.

## 4. Discussion

Nucleic acid vaccine, also known as third-generation vaccine, consists of a specific nucleic acid fragment encoding bacterial or viral antigens and gets taken up by the cells of an immunized species to induce an immunologic response. During the COVID-19 coronavirus pandemic, the field of nucleic acid vaccination has developed rapidly, along with the fields of adjuncts, such as delivery vectors assisting nucleic acid to enter cells. Since the discovery of the first CPP, CPPs have been considered as a significant delivery vehicle to transport a variety of cargo into the cells.

Proper bioinformatic approaches can improve the speed and accuracy of CPP screening and evaluation (Su et al., 2020). Hence, in the current study, we took advantage of multiple recently published bioinformatic tools and analyzed peptide P1’s physical-chemical properties, secondary and three-dimensional structures, cytotoxicity, immunogenicity, as well as CPP characterization through web server. We compared the prediction probabilities and scores and reevaluated our prediction through different algorithms. Meanwhile, we also predicted the sensitivity of peptide P1 with peptide truncation and single mutation, interestingly, we found that peptide P1 is sensitive in the two ends, and amino acids of position at 1, 2, 4–6, 10, 21, 22, 24, 25 are crucial for the penetration efficiency of peptide P1 because of the basic residue’s characteristics, although it still have positive charged residues in position 11 and 14. Thus, these data suggested that penetration efficiency may be affected by the charge of the residues and the location as well.

Our study may provide a new perspective for peptide research scientists on using appropriate prediction tools to suit their purposes. Bioinformatic screening and identification pipeline of CPPs and other functional peptides can contribute to the development and acceleration of the applications of peptide-based delivery systems.

Our bioinformatic prediction and wet-lab validation data suggest that peptide P1 derived from MARCKS protein phosphorylation site domain is a new CPP. We found that peptide P1 can efficiently internalize into various cell lines in a concentration-dependent manner. Receptor-mediated endocytosis pathway is the major mechanism of P1 penetration, although direct penetration of P1 is also involved. Peptide P1 has low cytotoxicity in cultured cell lines and mouse red blood cells. Furthermore, peptide P1 not only can enter into cultured cells itself, but also can interact with plasmid DNA and mediate plasmid DNA functional delivery.

In conclusion, we identified a new cell penetrating peptide P1 derived from MARCKS protein through *in silico* prediction and wet-lab experimental validation of cell-penetrating ability, and we found that peptide P1 can efficiently mediate plasmid DNA functional delivery into cultured cells, even in hard-to-transfect cells.
